# The prevalence of gastrointestinal symptoms and cobalamin deficiency in patients with chronic urticaria

**DOI:** 10.1186/s13223-023-00771-w

**Published:** 2023-02-24

**Authors:** Armin Abadeh, Sari M. Herman, Rupert Abdalian

**Affiliations:** 1 Department of Medicine, University of Toronto, 2703-488 University Avenue, Toronto, ON M5G0C1 Canada; 2 North York Medical Group, Toronto, ON Canada; 3 North York General Hospital, Toronto, ON Canada

**Keywords:** Urticaria, CSU, Gastrointestinal symptoms, Cobalamin deficiency, Vitamin B12

## Abstract

**Background:**

There is a paucity of studies reporting the presence of systemic symptoms and micronutrient deficiency in patients with chronic urticaria, and these data are lacking in a Canadian population.

**Objective:**

To report the prevalence of gastrointestinal symptoms and vitamin B12 (cobalamin) deficiency in a Canadian patient population diagnosed with chronic urticaria.

**Methods:**

A retrospective chart review of 100 adult patients with chronic urticaria was conducted. Demographic characteristics, medications, presence of gastrointestinal symptoms, and laboratory findings were abstracted from electronic medical records.

**Results:**

Seventy percent of patients with chronic urticaria reported experiencing gastrointestinal symptoms. The most common symptom identified was gastroesophageal reflux (42%). Vitamin B12 (cobalamin) deficiency, defined as serum vitamin B12 level ≤ 250 pmol/L, was identified in 31.7% of the patients. Among those patients with urticaria and vitamin B12, 68% reported gastrointestinal symptoms.

**Conclusions:**

This is the first study to provide data on the high prevalence of gastrointestinal symptoms and vitamin B12 (cobalamin) deficiency in a Canadian population diagnosed with chronic urticaria. Early recognition and management of systemic symptoms and micronutrient deficiency may lead to a more comprehensive approach to management of these patients.

*Trial registration* Not applicable

## Introduction

Chronic urticaria (CU) is defined as the occurrence of wheals, angioedema, or both for more than 6 weeks, with daily or almost daily signs and symptoms, or an intermittent/recurrent course [1]. The lifetime prevalence of this condition is estimated to be 1.4% [2].

Current literature suggests that extracutaneous symptoms including GI symptoms, arthralgias, headache and fatigue can be found in over a third of the patients with chronic urticarial [3]. The most common GI symptoms experienced by these patients, such as functional dyspepsia and gastroesophageal reflux are not life threatening, but nonetheless can lead to more serious health complications if left untreated [4]. GI symptoms not only have an enormous impact on the quality of life, work, and daily activities of affected individuals, they also impose substantial societal and economic costs [5]. These conditions are often under-recognized and remain untreated, which is concerning given their prevalence and the associated significant health and economic implications [6–8].

Untreated vitamin B12 (cobalamin) deficiency can lead to anemia and has been associated with a myriad of symptoms affecting multiple systems, including neurologic complications such as unsteady gait and paralysis, as well as depression and dementia [9]. In clinical practice, vitamin B12 (cobalamin) may be underdiagnosed as many symptoms are often overlooked [10]. There is also a lack of global consensus on the definition or a single measure of vitamin B12 (cobalamin) deficiency [11]. Overall, very few studies have examined the prevalence of vitamin B12 deficiency in patients with chronic urticarial [12–14].

Chronic urticaria seriously compromises the quality of life of patients due to its debilitating symptoms that can last for years [15, 16]. Highlighting that chronic urticaria involves other organ systems beyond the skin can lead to early recognition and a more comprehensive approach to the management of these patients which, can in turn, have an enormous impact on a patient’s quality of life, as well as societal and health care costs. The aim of this study was to report the prevalence of GI symptoms and vitamin B12 (cobalamin) deficiency in a Canadian patient population diagnosed with chronic urticaria.

## Methods

A retrospective review of the electronic medical records at an outpatient Allergy Clinic in Toronto, Ontario, Canada was conducted from April 2019 to February 2020. In this study, 100, consecutive, adult patients (age ≥ 18) with a diagnosis of chronic urticaria (ICD-9-CM Diagnosis Code 708) were included. This included patients with both chronic spontaneous and chronic inducible urticaria. Exclusion criteria included patients with a pre-existing diagnosis of mastocytosis.

Patient demographic data, clinical features including documented GI symptoms, medical comorbidities, medications, and laboratory results were abstracted from the electronic medical records of each patient meeting inclusion criteria for this study.

Vitamin B12 (cobalamin) was defined as serum level ≤ 250 pmol/L. Patients with a serum level of more than 250 pmol/L, excluding those on B12 supplementation at the time of initial consultation were categorized as having normal vitamin B12 (cobalamin) levels.

Statistical analyses were performed by using Microsoft Excel 2020. Pearson’s Chi-square test was used for categorical variables. p-value of < 0.05 was considered to be statistically significant.

Approval by the Institutional Review Board was obtained through the University of Toronto Research Ethics Board. This study met the criteria for a waiver of informed consent by the research ethics board.

## Results

### Patient demographics and baseline information

The average (mean) age of patients seen at the time of initial consultation was 44.8 years, with a standard deviation (SD) of ± 14.3. 24% of the patients were male, and 76% were female (Table 1). Patients taking H1-antagonist, H2-antagonist, proton pump inhibitor (PPI), anti-IgE therapy and/or oral vitamin B12 (cobalamin) replacement are outlined in Table 2. Ten patients reported taking vitamin B12 (cobalamin) supplementation at the time of initial consultation. Twenty patients had a co-existing autoimmune condition. The number of patients with each autoimmune condition is depicted in Table 3.

**Table 1:** Characteristics of study patients with chronic urticaria

	N = 100
Sex	
Male	24
Female	76
Age	
≤ 30	17
31–40	25
41–50	23
≥ 50	35

**Table 2:** Medication profile of study patients with chronic urticaria

	Number of patients
H1-antagonist	
Prescribed prior to consultation	79
Prescribed after allergy consultation	11
H2-antagonist	
Prescribed prior to consultation	3
Prescribed after consultation	14
PPI	
Prescribed prior to consultation	10
Prescribed after consultation	8
Anti-IgE	
Prescribed prior to consultation	1
Prescribed after consultation	7
Vitamin B12 supplementation	
Initiated prior to consultation	10
Initiated after consultation	7

**Table 3:** Prevalence of co-existing autoimmune conditions in the study population

Co-existing auto-immune conditions	Number of patients
Hypothyroidism	8
Diabetes Mellitus Type 1	3
Inflammatory bowel disease	3
Vitiligo	3
Alopecia areata	2
Psoriasis	1
Celiac disease	1

#### Gastrointestinal symptoms

Seventy patients reported experiencing GI symptoms at initial consultation or follow-up visits (Table 4). The most common symptoms were gastroesophageal reflux (n = 42; 60%), epigastric pain (n = 26; 37%), cough, dysphonia, or excessive throat clearing (n = 19, 27%), dyspepsia (n = 15, 21%), and abdominal bloating (n = 14, 20%).

**Table 4:** Frequency of gastrointestinal symptoms in study patients

	Number of patients (N = 100)
Co-existing gastrointestinal symptoms	70
Gastroesophageal reflux	42
Epigastric pain/discomfort	26
Cough, hoarseness, excessive throat clearing	19
Dyspepsia	15
Abdominal bloating	14
Dysphagia	10
Nausea	8
Diarrhea	6
Globus sensation	3
Vomiting	2

### Vitamin B-12 (cobalamin) deficiency

From the 100 patients included in this study, 92 patients had a measured serum vitamin B12 (cobalamin) level. The average serum level in these patients was 361 pmol/L (including data from patients who reported taking vitamin B12 supplementation). 27 patients had a serum level of ≤ 250 pmol/L and hence were classified as vitamin B12 (cobalamin) deficient. Excluding patients on B12 supplementation, 58 patients had a serum level > 250 pmol/L and were classified as having normal vitamin B12 levels. Among those patients with vitamin B12 (cobalamin) deficiency, 68% reported GI symptoms. In patients without identifiable vitamin B12 deficiency on measurement, 66% reported GI symptoms. Comparing the two groups, this difference was not statistically significant (p > 0.05).

### H. pylori infection

In this study, H. pylori infection status was available for 30 patients. Of these 30 patients, 6 patients (20%) were H. pylori positive based on either serology or gastric biopsy. Of the 18 patients with available H. pylori serology, 5 patients were positive. Of the 14 patients who underwent an endoscopy, 2 patients had confirmed infection on biopsy.

### Thyroid antibodies

Anti-thyroid peroxidase (TPO) was positive in 14 (22%) and anti-thyroglobulin (Tg) was positive in 16 (25%) of the 63 patients tested.

## Discussions

To our knowledge, this is the first study to provide data on the high prevalence of GI symptoms in patients with CU in a Canadian population. This finding was consistent with what has been reported in the literature internationally. For example, in an American study involving 155 patients with chronic spontaneous urticaria, more than 66% patients reported systemic complaints, with GI complaints being present in 26.2% of total study population [17]. In another study in Italy, the prevalence of GI complaints in patients with urticaria was reported as 44%, which was four-times higher compared to patients without hives [18]. It is interesting to note that the overall prevalence of GI symptoms (70%) in our study, exceeded what has been previously reported in American and European studies.

Various mechanisms have been discussed in the literature to explain the reported GI and other systemic complaints in patients with CU. The gradual and simultaneous increase in IgE and eosinophil levels may suggest the potential role of progressive increase in the activation of a Th2-like immune-inflammatory response in these patients [18]. Evidence also suggests that the number of mast cells may be increased in the stomach and duodenum in patients with chronic urticaria independent of the occurrence of GI symptoms [19]. Histamine can play a crucial role in the occurrence of GI symptoms in patients with urticaria as its significant effects on the GI tract have been well described [20]. In a study by Guida et al. plasma histamine concentrations were found to be higher in chronic spontaneous urticaria patients which may explain the presence of histamine-mediated symptoms including increase in stomach acid production [21].

This study also reveals the higher prevalence of vitamin B12 (cobalamin) deficiency in patients with CU which is consistent with previous reports. A study by Wu et al. [13] compared 176 patients with chronic urticaria with 1320 normal individuals and reported a significantly higher prevalence of vitamin B12 deficiency in patients with CU. Similarly, another comparative study which included 42 patients with CU and 19 healthy individuals also reported significantly lower B12 levels in patients with CU [12]. Vitamin B12 (cobalamin) deficiency was also observed in one-third of patients (n = 33) with CU compared with the control group in a study by Mete et al. [14]. The higher prevalence of vitamin B12 deficiency in these patients is likely multifactorial. The association between chronic urticaria and other GI diseases such as celiac disease has been previously reported [22]. In these cases, vitamin B12 (cobalamin) deficiency could be attributed to the disturbances in normal GI functioning, leading to malabsorption [23, 24]. Furthermore, prolonged use of PPI and antihistamines in this patient population is also associated with vitamin B12 deficiency [25]. In this study, at the time of initial consultation, 79% of our patients were using second-generation H1-antihistamines and 10% were using PPI. In addition to these factors, several case–control and prospective cohort studies have shown that patients infected with H. pylori, have lower vitamin B12 levels when compared to control groups. This becomes even more important as a higher prevalence of H. pylori infections has been reported in patients with CU. Multiple studies have reported vitamin B12 deficiency in patients with H. pylori infection [26, 27].

Although effective therapies for CU have become available in recent years, treatment outcomes are often concentrated on the number of wheals, severity of pruritus, and quality of life, and do not address the extracutaneous manifestations of this condition, or their impact on quality of life [28]. Given the high prevalence of GI symptoms and vitamin B12 (cobalamin) deficiency in patients with CU in our Canadian population, a systematic inquiry of GI symptoms may be applied in those with CU. Likewise, vitamin B12 level measurement may be contemplated in those managing patients affected by longstanding CU. If identified, it would be prudent to search for an underlying cause vitamin B12 (cobalamin) deficiency including such as chronic H. pylori infection, medication use (e.g. PPI, H2 antihistamine, metformin), underlying medical disease (e.g. celiac disease, inflammatory bowel disease), inadequate replacement following surgery (e.g. gastric resection, small bowel resection or bariatric surgery), and dietary restriction (e.g. vegans).

Limitations of this study include its retrospective nature, the inherent subjective evaluation of medical records and the lack of additional investigations such as anti-parietal antibodies, methylmalonic acid and homocysteine levels, due to data unavailability. Given the wide range of symptoms affecting multiple systems in those patients presenting with this deficiency, urticaria may be considered in itself a manifestation of vitamin B12 (cobalamin) deficiency and/or a flag for the presence of this deficiency. Future systematic studies to explore the involvement of multiple micronutrients deficiency, microbial flora, and the role of various gastrointestinal motility disorders may be valuable.

## Data Availability

The datasets during and/or analysed during the current study available from the corresponding author on reasonable request.

## Abbreviations

CU: Chronic urticaria
GI: Gastrointestinal
H. pylori: Helicobacter pylori
